# Acute Radiation Esophagitis After Chemoradiotherapy Demonstrating “Tube‐Like” FDG Uptake on PET‐CT: Case Report and Literature Review

**DOI:** 10.1002/cnr2.70365

**Published:** 2025-10-16

**Authors:** Tatsuyuki Kawahara, Nobuaki Ochi, Kensuke Matsuzaki, Hirohito Kirishi, Yusuke Sunada, Ayaka Mimura, Naruhiko Ichiyama, Yoko Kosaka, Yasunari Nagasaki, Hidekazu Nakanishi, Hiromichi Yamane, Nagio Takigawa

**Affiliations:** ^1^ Department of General Internal Medicine 4 Kawasaki Medical School Okayama Japan

**Keywords:** lung cancer, positron emission tomography, radiation esophagitis

## Abstract

**Background:**

Radiation esophagitis (RE) is a frequent complication of concurrent chemoradiotherapy (CRT) for non‐small cell lung cancer (NSCLC). However, its acute‐phase imaging features on positron emission tomography‐computed tomography (PET‐CT) are rarely described in detail. Early identification of such findings may help differentiate RE from other causes of esophageal fluorodeoxyglucose (FDG) uptake.

**Case:**

We report a case of a 75‐year‐old man with stage IIIA NSCLC who underwent CRT with cisplatin and S‐1 alongside intensity‐modulated radiation therapy (IMRT). During the acute phase, PET‐CT revealed an unusual tube‐like pattern of FDG uptake in the esophagus (SUVmax = 5.5), which subsequently resolved. The patient later developed chronic RE presenting as dysphagia, which gradually improved with conservative treatment.

**Conclusion:**

This case highlights an atypical tube‐like FDG uptake pattern on PET‐CT during acute RE and underscores the need for awareness of this finding. A review of the literature suggests potential overlap with imaging features of chemotherapy‐induced or Candida esophagitis. Larger studies are warranted to evaluate whether such PET‐CT findings may serve as early markers for acute RE and predictors of chronic complications.

## 1 Introduction

Lung cancer remains the leading cause of cancer‐related mortality worldwide. Approximately 22% of patients present with locally advanced NSCLC, for which concurrent chemoradiotherapy (CRT) is the standard of care in patients not eligible for surgery [1]. Despite advances in treatment, the prognosis remains poor, with a median survival of 16–27 months, and 2‐ and 5‐year survival rates of 39%–60% and at most 20%, respectively [1, 2, 3]. While CRT improves survival, it is also associated with both acute and late toxicities affecting normal tissues. Radiation esophagitis (RE) is one of the most common and clinically significant complications, presenting acutely during or soon after treatment and, in some cases, progressing to chronic sequelae such as strictures and dysphagia [4, 5, 6].

Characteristic imaging features of RE on [18F]‐fluorodeoxyglucose (FDG) positron emission tomography‐computed tomography (PET‐CT), especially in the acute phase, are rarely reported. Lang et al. described FDG PET‐CT manifestations of acute esophagitis in oncologic settings, including post‐CRT [5], but few case reports have detailed a distinct “tube‐like” uptake pattern. Similar esophageal FDG uptake has been reported in chemotherapy‐induced reflux esophagitis [7] and in Candida esophagitis [8, 9, 10]. Differentiating these entities is clinically important, as management and prognosis differ.

Here, we present a case of acute RE after CRT for NSCLC with a rare “tube‐like” FDG uptake pattern on PET‐CT, and we review the literature on similar imaging appearances and their differential diagnoses.

## 2 Case Report

**FIGURE 1:**
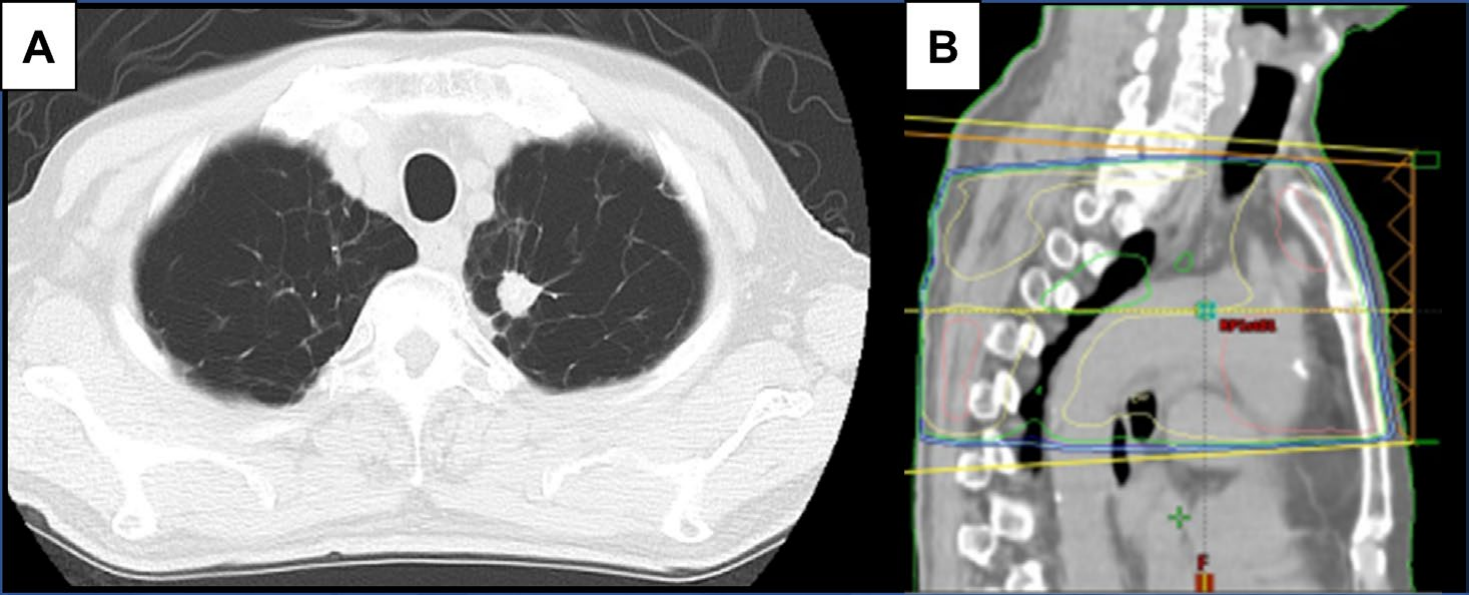
(A) Chest CT shows emphysematous changes and a 2.5 cm mass in the left upper lobe. (B) Intensity‐modulated radiation therapy was administered at a total dose of 60 Gy in 30 fractions over 6 weeks, targeting the primary tumor and ipsilateral mediastinal lymph nodes.

**FIGURE 2:**
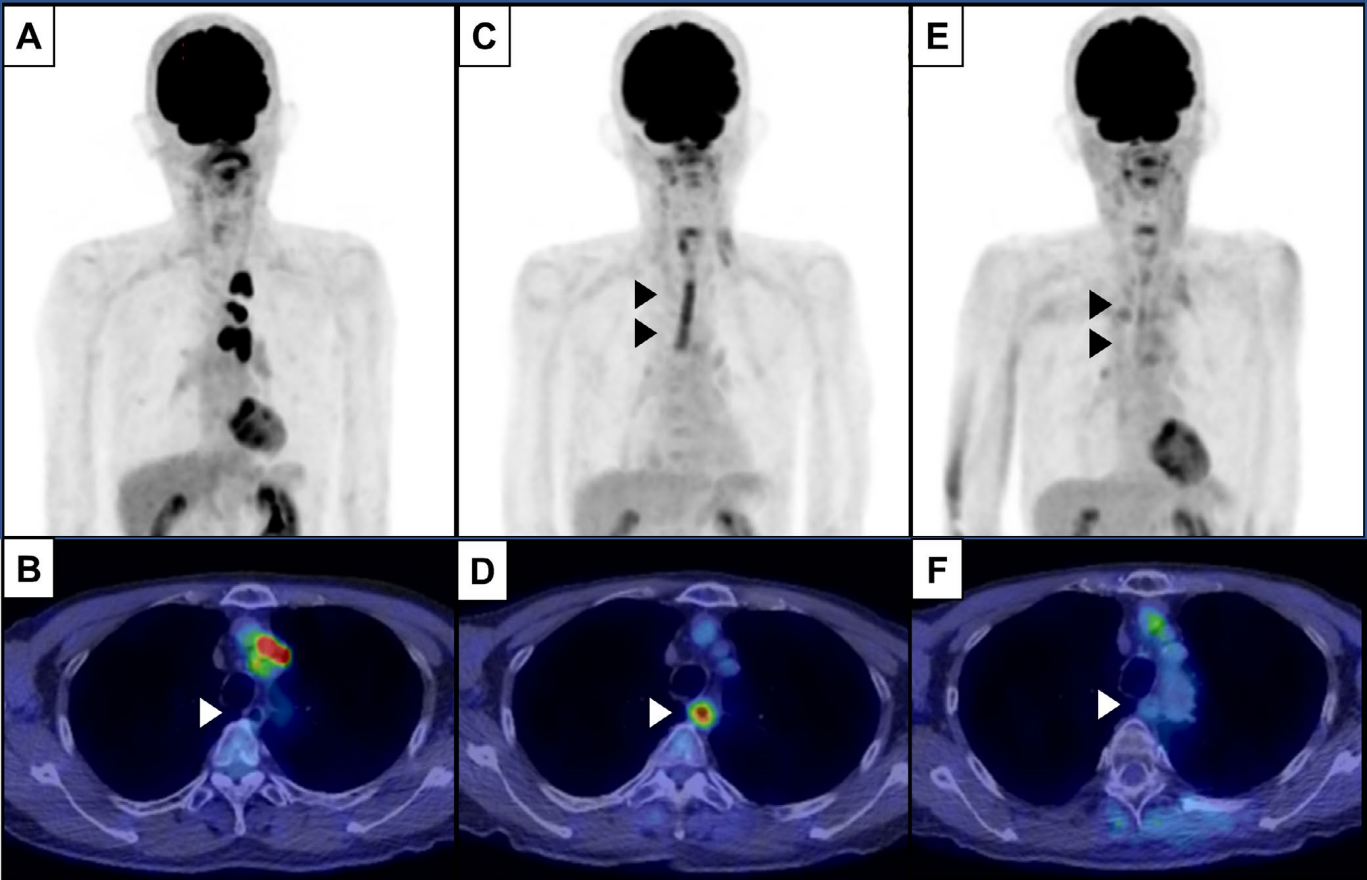
(A, B) PET imaging shows increased [18F]‐FDG uptake in the tumor located in the left upper lobe and the ipsilateral mediastinal lymph nodes. (C, D) One month after completion of definitive chemoradiotherapy, PET revealed abnormal FDG uptake (SUVmax = 5.5) along the esophagus in a tube‐like pattern (arrowheads). (E, F) Seven months after treatment, the abnormal esophageal FDG uptake had completely resolved (arrowheads).

**FIGURE 3:**
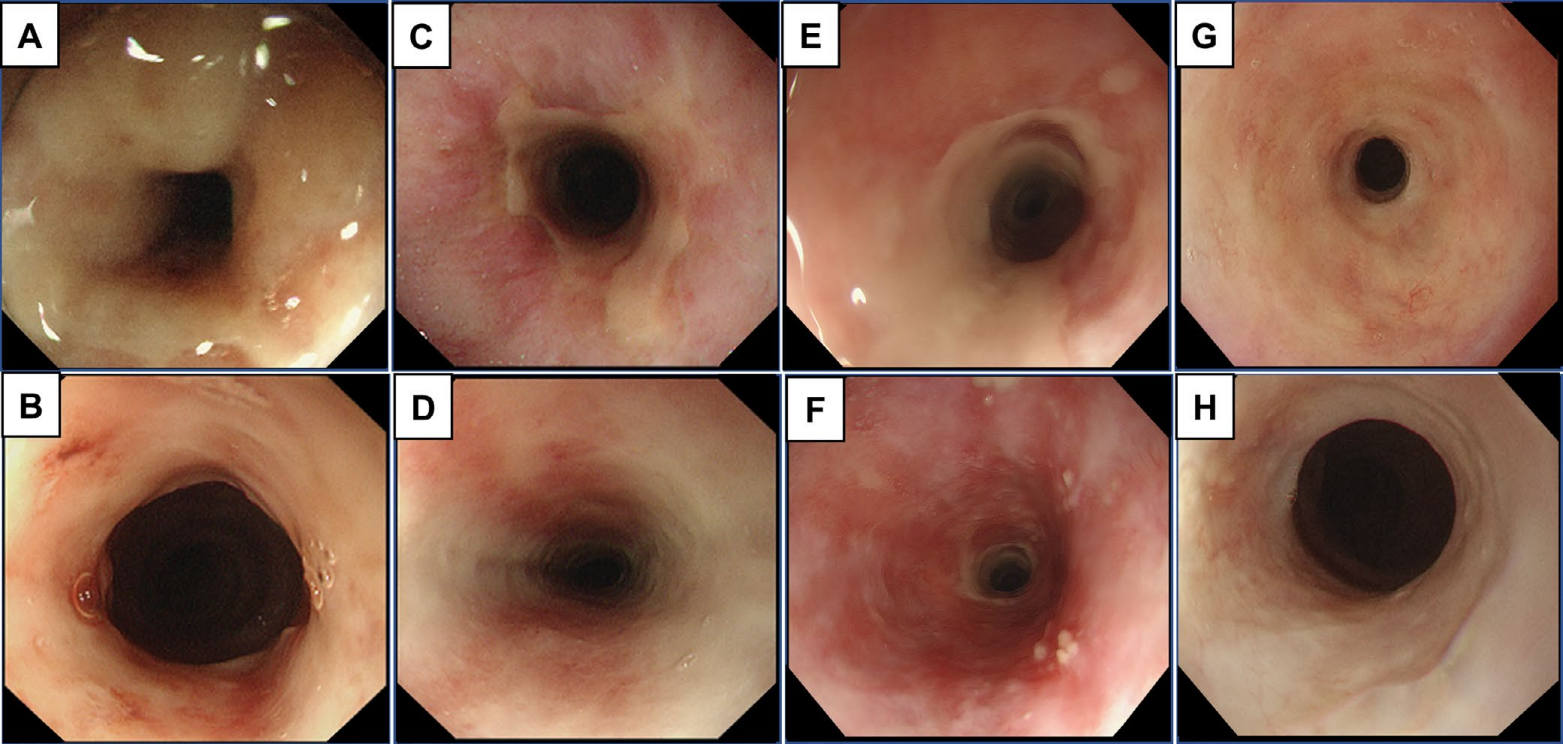
Upper gastrointestinal endoscopy was performed to evaluate the onset and progression of esophageal mucosal injury. (A, B) One month after treatment, erosions were observed at the esophageal inlet (A), and a well‐demarcated ulcer with stricture lacking mucosal lining was detected at 20–27 cm from the incisors. Passage of a 10.2‐mm diameter fiberscope was not possible (B). (C, D) At 4 months post‐treatment, both the erosions and ulcers had improved. (E, F) However, 3 months later, a moderate esophageal stricture and worsening of erosive lesions were again observed at 25–30 cm from the incisors. (G, H) At 16 months after treatment, mild residual stenosis remained, but no re‐exacerbation was observed.

A 75‐year‐old man with a 40 pack‐year smoking history presented with cough and hoarseness in February 2024 at Kawasaki Medical School General Medical Center. Chest CT revealed emphysematous changes and a 2.5 cm mass in the left upper lobe (Figure 1A). A diagnosis of lung adenocarcinoma was confirmed by transbronchial biopsy. FDG PET‐CT showed high uptake in the pulmonary nodule and mediastinal lymph nodes, consistent with stage IIIA disease (cT2N2M0) (Figure 2A). He underwent concurrent thoracic radiotherapy delivering 60 Gy in 30 fractions using intensity‐modulated radiation therapy (IMRT), and two cycles of combination chemotherapy with cisplatin and S‐1 (tegafur–gimeracil–oteracil potassium), consisting of cisplatin 60 mg/m^2^ intravenously on days 1 and 8, and S‐1 40 mg orally twice daily for 14 days [11]. IMRT was delivered over 6 weeks, targeting the primary tumor and ipsilateral mediastinal lymph nodes (Figure 1B). Two weeks after completing CRT, the patient developed anterior chest pain. PET‐CT demonstrated complete metabolic remission of the primary lesion, but revealed tube‐like longitudinal FDG uptake in the mid‐esophagus (SUVmax = 5.5) (Figure 2B). Esophagography confirmed severe esophageal stricture associated with esophagitis (Figure 3A,B). Biopsy ruled out Candida esophagitis. Conservative therapy with sodium alginate improved symptoms and imaging findings over several months (Figure 3C,D). At 7 months post‐CRT, FDG uptake had resolved (Figure 2C), but chronic RE with dysphagia persisted, limiting solid food intake. Endoscopic dilation and surgery were considered, but conservative management was chosen due to rupture risk. At 16 months post‐CRT, endoscopy showed no recurrent stricture (Figure 3G,H), and swallowing function had improved to a normal diet. A timeline summarizing the patient's clinical course is presented in Table 1.

**TABLE 1:** Timeline of the patient's clinical course.

Timeline	Clinical events and intervention	PET‐CT findings	Endoscopic findings of upper gastrointestinal endoscopy
Nov 2018	Referred to otolaryngology at a local clinic with hoarseness		
Jan 2019	Diagnosed with large cell carcinoma of the lung (cT2aN2M0, stage IIIA)	Abnormal FDG uptake in the left upper lobe tumor and ipsilateral mediastinal lymph node (Figure 2A,B)	
Feb 2019	Underwent concurrent chemoradiotherapy: CDDP + S‐1 for 2 cycles, combined with RT (60 Gy/30 fractions)		
Mar 2019	Developed dysphagia; radiation esophagitis was suspected. Sodium alginate was administered, and radiotherapy was discontinued		
Apr 2019	Worsening dysphagia; diagnosed with radiation esophagitis	Tube‐like esophageal FDG uptake (SUVmax = 5.5) (Figure 2C,D)	Severe esophageal ulcer and stricture at 20–27 cm from the incisors (Figure 3A,B)
Jun 2019	Improvement confirmed on follow‐up		Partial resolution of esophageal erosion and stricture (Figure 3C,D)
Sep 2019	Recurrence of dysphagia; diagnosed with chronic radiation esophagitis	Disappearance of FDG uptake in the esophagus (Figure 2E,F)	
Oct 2019	Balloon dilation was considered, but conservative management was chosen due to patient preference and procedural risk		
Sep 2020	Dysphagia resolved	No recurrent esophageal FDG uptake was observed	Residual stenosis without exacerbation (Figure 3G,H)

Abbreviations: CDDP, cisplatin; FDG, fluorodeoxyglucose; PET‐CT, positron emission tomography‐computed tomography; RT, radiation therapy; S‐1, tegafur–gimeracil–oteracil potassium.

## 3 Discussion

In this report, we describe an extremely rare PET‐CT finding of RE. Although the patient with locally advanced NSCLC achieved metabolic complete remission after CRT, a tube‐like pattern of FDG uptake was observed at the site corresponding to the esophagitis.

Radiotherapy exerts its effects by generating photon‐induced free radicals that damage cellular DNA and impair cellular replication. While normal cells are generally capable of recovering from this damage, cancer cells often lack adequate DNA repair mechanisms. However, surrounding normal tissues are also affected, and the resulting toxicities are typically classified as acute, subacute, or late effects [4]. A recent analysis of modern CRT series—including secondary analyses of large cooperative‐group trials and contemporary IMRT series—shows that the incidence of grade ≥ 3 acute esophagitis is substantially lower than previously reported, generally in the low‐teens percent range (approximately 13%–15% in some contemporary analyses). The peak of symptomatic esophagitis still occurs within 1–2 months after radiotherapy [12]. This finding replaces the older high estimates and reflects current IMRT techniques and supportive‐care practices.

Acute toxicities are most commonly observed during the treatment period and usually resolve within one to two weeks post‐therapy. Because radiotherapy targets dividing cells, normal tissues with high cellular turnover—such as the mucosal linings of the oral cavity, esophagus, and skin—are particularly susceptible, leading to adverse effects such as mucositis, esophagitis, and dermatitis [4].

The total volume of esophagus and a higher dose per fraction (2 Gy vs. 1.8 Gy) has been associated with an increased risk of Grade ≥ 2 acute esophagitis [12]. IMRT, which allows for concave dose distributions around organs at risk, requires meticulous planning to minimize esophageal toxicity [13]. Subacute toxicities, such as radiation pneumonitis, typically emerge within one to three months after treatment completion. Recent studies show that most symptomatic cases present within 6–12 weeks, although late presentations up to 6 months have been reported. Late toxicities, such as esophageal stricture and dysphagia, are less frequent but clinically important; contemporary series report variable latency—commonly within about 3–9 months and often between 4 and 12 months after treatment—while isolated cases and registry/guideline reports document onset beyond 1 year in selected patients [6, 14].

In a review by Umsawasdi et al. [15], the incidence of esophageal stricture was reported to be 0.1% (2 of 2014 cases) in patients treated with radiotherapy alone, compared to 0.8% (11 of 1345 cases) in those treated with CRT. Moreover, abnormal esophageal motility may present as early as three to four weeks after RT alone, and as early as 1 week following the initiation of concurrent CRT [16]. Combined modality therapy generally results in earlier onset and more severe mucosal toxicities compared to radiotherapy alone. While grade 3 or higher esophagitis was not reported in the phase II study of cisplatin plus S‐1 with RT [11], physicians should be aware that grade 3 esophagitis, as observed in the present case, may still occur.

Although RE is a well‐recognized complication of thoracic radiotherapy, its characteristic imaging features on FDG PET‐CT have been infrequently described. In the present case, FDG PET‐CT demonstrated a rare diffuse “tube‐like” uptake pattern extending along the entire esophagus, with only a few similar patterns reported in the literature. Bural et al. reported a similar diffuse uptake pattern in a patient with reflux esophagitis after chemotherapy but did not use the term “tube‐like” to describe the finding [17]. Lang et al. [5], in their review of FDG PET‐CT manifestations in the thoracic region after oncologic treatment, described linear uptake in acute esophagitis but without defining a tubular configuration. Notably, Ulaner et al. [18] reported similar findings in a patient with malignant lymphoma. This tube‐like pattern is hypothesized to result from irradiation of a broad esophageal field, in which the inflammatory response primarily involves the submucosal and muscularis layers, ultimately leading to fibrosis.

Differential diagnosis for diffuse esophageal FDG uptake includes reflux esophagitis, drug‐induced esophagitis (e.g., bisphosphonates, doxycycline), infectious esophagitis of viral or fungal origin, and diffuse infiltrative carcinoma [5, 7, 8, 9, 10]. Educationally, correlation with patient history, symptom onset, treatment exposure, and endoscopic findings is essential to avoid misinterpretation and to guide appropriate management.

In the present case, characteristic endoscopic findings of Candida esophagitis, such as adherent white plaques, were not observed, and the lesion was confined to the radiation field. Furthermore, histopathological examination excluded the diagnosis of Candida esophagitis. In previous reports, similar diffuse or multifocal FDG uptake patterns have been reported in Candida esophagitis [8, 9, 10], most of which occurred in immunocompromised patients after chemotherapy or chemoradiation. Compared with our case, these reports often demonstrated patchy or multifocal mucosal involvement with characteristic endoscopic white plaques, whereas our patient showed a continuous “tube‐like” uptake limited to the irradiated esophageal segment, without endoscopic features of Candida infection.

## 4 Conclusion

We present an instructive case of acute and chronic RE following concurrent chemoradiotherapy, characterized in the acute phase by a rare “tube‐like” FDG uptake pattern on PET‐CT (SUVmax = 5.5), which resolved over time. This case highlights the importance of recognizing uncommon imaging manifestations of RE and the necessity of vigilant long‐term follow‐up, as late complications such as esophageal stricture can occur even after acute symptoms resolve. The observed “tube‐like” uptake may serve as a potential imaging surrogate for acute RE; however, validation of this finding in larger patient cohorts is warranted to determine its diagnostic reliability and to explore whether early detection of such a pattern could help predict progression to chronic esophagitis or stricture.

## Author Contributions

**Tatsuyuki Kawahara:** conceptualization, writing – original draft. **Nobuaki Ochi:** conceptualization, project administration, supervision, writing – review and editing. **Kensuke Matsuzaki, Hirohito Kirishi, Yusuke Sunada, Ayaka Mimura, Naruhiko Ichiyama, Yoko Kosaka, and Yasunari Nagasaki:** review and editing. **Hidekazu Nakanishi, Hiromichi Yamane, and Nagio Takigawa:** supervision, writing – review and editing.

## Ethics Statement

The ethical approval was waived by the Institutional Review Board of our hospital as this is a case report.

## Consent

Written informed consent was obtained from the patient for publication of this case report and accompanying images.

## Conflicts of Interest

The authors declare no conflicts of interest.

## Data Availability

The authors have nothing to report.
